# Decompression with or without fusion in degenerative adjacent segment stenosis after lumbar fusions

**DOI:** 10.1007/s10143-022-01875-4

**Published:** 2022-10-04

**Authors:** Anton Früh, Patrick Leißa, Dimitri Tkatschenko, Peter Truckenmüller, Lars Wessels, Peter Vajkoczy, Simon Bayerl

**Affiliations:** 1 Department of Neurosurgery, Charité - Universitätsmedizin Berlin, Corporate Member of Freie Universität Berlin, and Humboldt-Universität Zu Berlin, and Berlin Institute of Health, Berlin, Germany; 2 Universität Witten-Herdecke, Witten, Germany

**Keywords:** Lumbar adjacent segment stenosis, Decompression, Fusion surgery

## Abstract

Adjacent segment stenosis can occur after lumbar fusion surgery, leading to significant discomfort and pain. If further surgeries are required, the choice of the operative technique is an individual decision. In patients without over instability, it is still uncertain whether patients with adjacent spinal stenosis should be treated like primary lumbar spinal stenosis via decompressive surgery alone or with decompression and fusion. This is a retrospective analysis with prospective collected data. We included patients with adjacent segment stenosis after lumbar fusion. Patients with spinal deformity and/or obvious instability and/or significant neuroforaminal stenosis were excluded. All patients were divided into two groups according to the surgical technique that has been used: (a) treated via microsurgical decompression (MDG), (b) decompression and fusion of the adjacent segment (FG). Treatment decision was at discretion of the surgeon. Primary outcome was the need for further lumbar surgery after 1 year. In addition, patient reported outcome was measured via numerical rating scale (NRS), SF-36, Oswestry disability Index (ODI), Pittsburgh Sleep Quality Index (PSQI), and General Depression Scale before and after 1 year after surgery. In a further follow-up, need for additional lumbar surgery was redetermined. Total study population was 37 patients with a median age of 72 years. A total of 86.1% of patients suffered from a proximal adjacent segment stenosis and most common level was L3/4 (51.4%). A total of 61.1% of included patients developed adjacent segment stenosis after fusion of one single lumbar segment. Eighteen patients were included in MDG and 19 patients in FG. Both groups benefited from surgical interventions and there was no significant difference concerning pain, pain associated disability, sleeping, life quality, and mood after 1 year or the need of follow-up surgeries 1 year after primary fusion (5 in MDG vs. 5 in FG, *p* = 0.92) and at the second follow-up with a median time after surgery of 30 months (6 in MDG vs. 7 in FG, *p* = 0.823). Duration of surgery and hospital stay was significant shorter in MDG. There was no difference concerning operative complications rate. Both groups improved significantly in pain associated disability index, pain in motion, and concerning the sleeping quality. The present study indicates that decompression may not be inferior to decompression and fusion in patients suffering from degenerative adjacent segment stenosis without obvious signs of instability, deformation, and neuroforaminal stenosis after lumbar fusion in short-term follow-up. Due to significant shorter time of surgery, a pure microsurgical decompression may be a sufficient alternative to a decompression and fusion, particular regarding old age of this patient cohort.

## Introduction

Lumbar fusions are well established procedures for treatment of various lumbar diseases [1, 2]. Despite initially satisfying clinical results, adjacent segment degeneration is one potential long-term complication [3]. Thereby, radiographic evidence of adjacent segment degeneration can be found in up to 30%, whereby incidence of patients with clinical symptomatology is significantly lower [4, 5].

In patients suffering from primary lumbar spinal stenosis, decompression surgery with fusion did not result in better clinical outcomes than decompression surgery alone [6]. Furthermore, decompression alone strategies lead to shorter durations of surgeries and length of hospital stays [7].

In contrast, management of patients with symptomatic spinal stenosis adjacent to previous lumbar fusions remains controversial [8]. It is still uncertain whether patients with adjacent spinal stenosis without obvious instability should be treated via decompressive surgery alone or decompression and fusion.

Thus, the aim of our study was to generate further evidence concerning the treatment of patients suffering from symptomatic adjacent spinal stenosis after previous lumbar fusions.

## Methods

### Patient cohort

This is a retrospective single center study performed at our tertiary medical center. It was approved by the local ethics committee of Charité University Hospital (ethical approval number: EA2/093/13). All patients over the age of 18 years were eligible. We included patients with adjacent segment stenosis after treatment with posterior fusion and pedicle screw fixation between L1 and S1. Thereby, patients initially treated with single, two level, and multilevel fusions up to L1 using pedicle screws were included into the present study. All patients had to have radiographic evidence of spinal stenosis adjacent to previous lumbar fusions verified by magnetic resonance imaging (MRI). Due to our clinic standard, all patients received CT scans, long standing and flexion–extension radiographs. Patients with spinal deformity, fractures, and/or obvious instability, indicated by slippage above 3 mm of vertebral bodies in flexion–extension radiographs, were excluded. Furthermore, all patients with foraminal stenosis of grade 3 on MRI scans according to the classification of Lee et.al. [9] were not included.

### Intervention

**Fig. 1:**
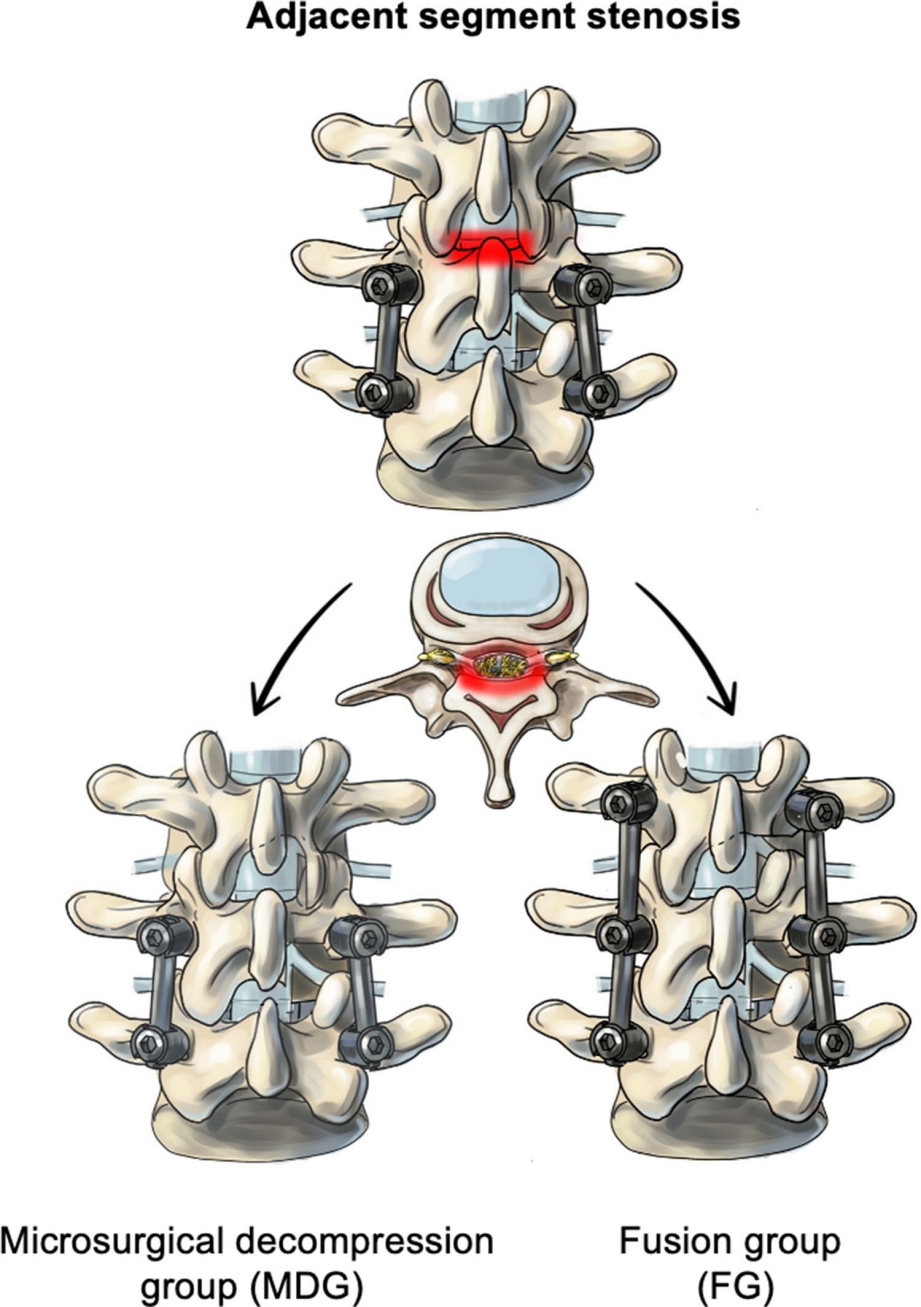
Different surgical techniques of the study. MDG, microsurgical decompression group. FG, fusion group

All patients were divided into two groups according to the surgical technique that has been used: (a) treated via microsurgical decompression (MDG), (b) decompression and fusion of the adjacent segment (FG). Thereby, it was an individual decision of the surgeon at discretion of the surgery which strategy was chosen. All patients with adjacent segment stenosis after lumbar fusion in this study presented with severe signs of degeneration in the adjacent segment. Degeneration went along with osteochondrosis, loss of vertebral disc height, facet joint hypertrophy, and ligamental hypertrophy. All these degenerative changes may indicate a micro-instability. Therefore, in our tertiary center, we initially mainly performed fusion surgery in these patients. However, because of our subjective good outcome in patients that refused fusion and were treated with decompression only, it was an individual decision of the surgeon whether a fusion was performed in patients with no clear instability. The different surgical techniques are illustrated in Fig. 1.

### Outcomes

At hospital admission, all clinical information, patient characteristic, symptoms, and outcome parameters were documented in a prospective and standardized way using institutional routine questionnaires. Patients were included prospectively into a spine registry. Patient reported outcome was measured according to our routinely clinical standard via numerical rating scale (NRS) values on an 10-point pain scale at rest and in motion, quality of life SF-36 form [10], disability in activities of daily life via Oswestry disability Index (ODI) [11], sleep quality via Pittsburgh Sleep Quality Index (PSQI) [12], and mood states via short form of the General Depression Scale (ADS-K) [13]. Further preoperative radiographic measurements were performed using Phoenix-PACS MERLIN diagnostic software. In detail, lumbar lordosis angle, segmental lordosis at the fusion levels, and pelvic incidences were measured in long standing x-rays. Disc heights and diameters of spinal canals were measured in preoperative MRI scans. For osteoporosis assessment, hounsfield units (HUs) of adjacent vertebral body were determined in preoperative CT-scans with 1 mm slices [14]. Therefore, an oval region of interest was placed over axial image of the vertebral body of the adjacent corporal body. In addition, duration of surgery and hospital stay and incidence of dura leaks during surgery were recorded. According to our routine clinical follow-up, patients were revisited after 12 months and 2 to 3 years after surgery at our outpatient’s clinic and clinical and outcome parameters were routinely assessed according to prior admission. Primary outcome of the study was the need for further lumbar surgery within 1 year after surgery. Secondary outcomes of the study were clinical conditions of the patients after 1 year and the need for the following need of lumbar surgery.

### Statistics

Statistical analysis was performed with SPSS version 25 (IBM Corp), Microsoft Excel 2021, and GraphPad Prism 8.4.2. Discrete data were presented as count and percentage and analyzed by using chi-square test. Continuous data were presented as median and interquartile range (IQR) and compared using Mann–Whitney statistics. Two-sided *p*-values < 0.05 were taken to indicate statistical significance.

## Results

**Fig. 2:**
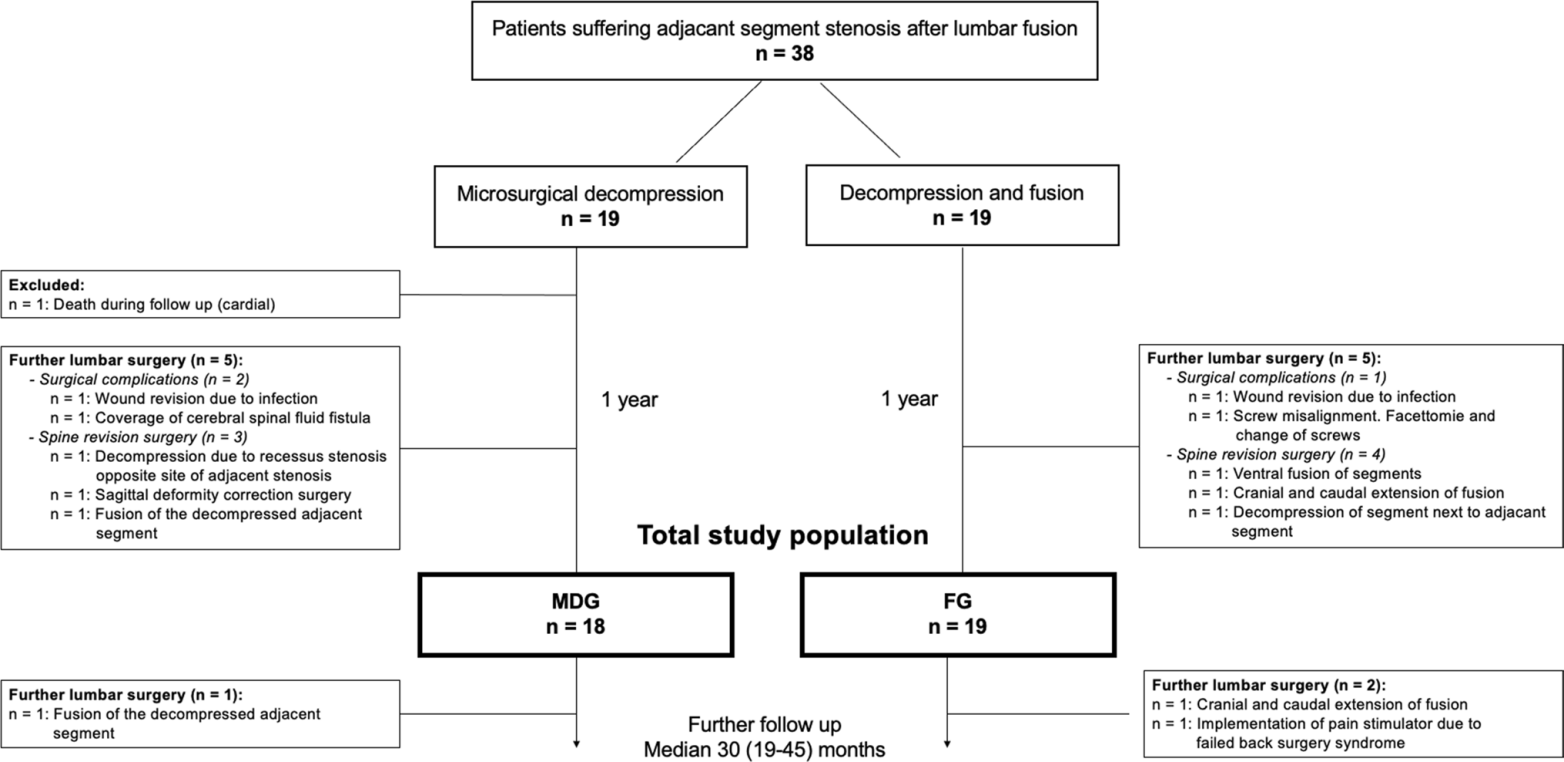
Study flow diagram. MDG, microsurgical decompression group. FG, fusion group

Initially, a total of 38 patients were enrolled in the present study. One patient was excluded during the follow-up period due to cardiac death. Therefore, total study population of this study was 37 patients. A detailed study flow diagram is provided in Fig. 2.

**Fig. 3:**
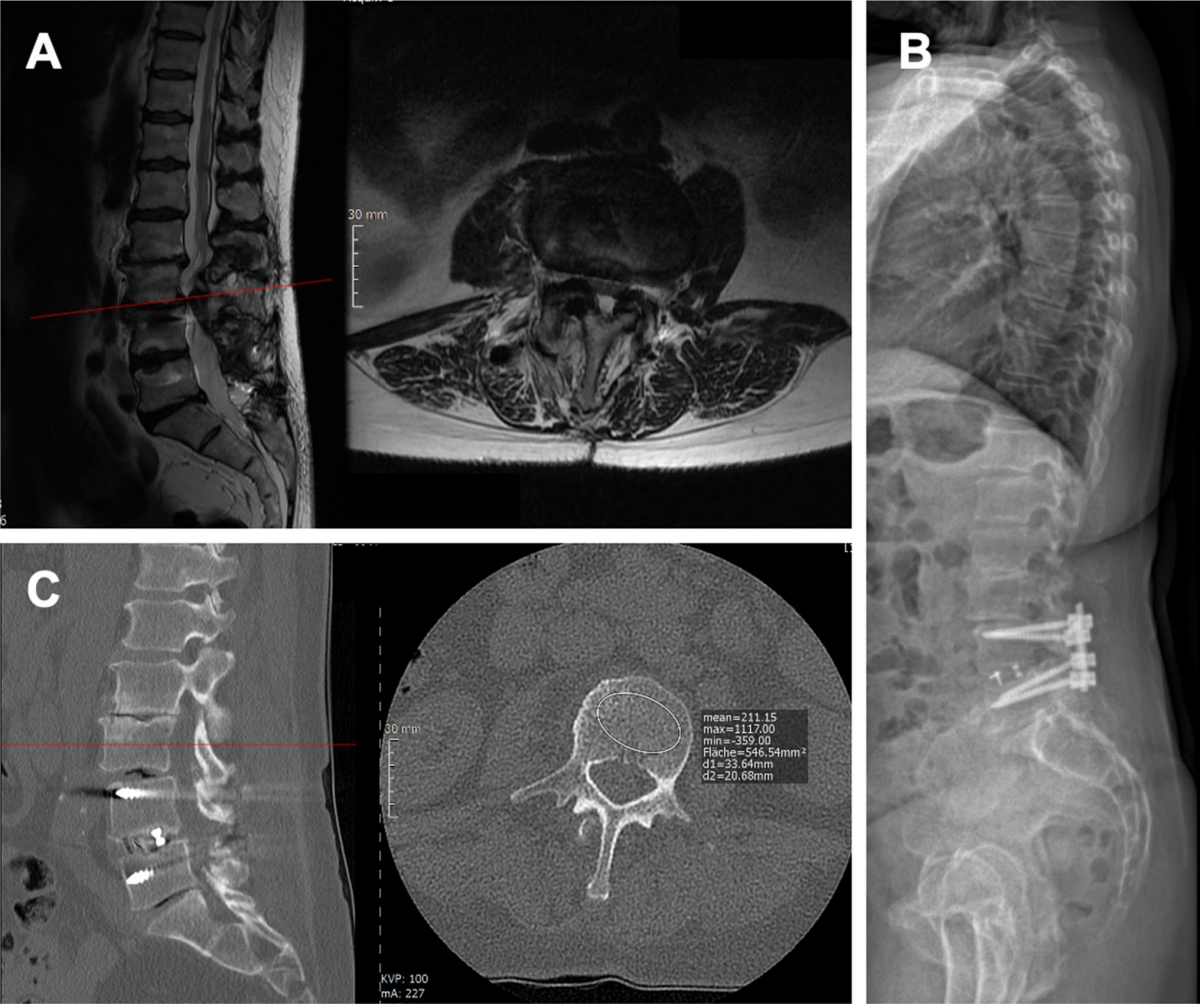
Radiological imaging of a typical patient. **A** MRI imaging. Left, sagittal. Right, axial; **B** long standing X-ray; **C** CT-scan. Left, sagittal. Right, axial

Radiological imaging of a typical patient is provided in Fig. 3. After fusion of L4/5 and a symptom free interval, this patient is suffering from claudication symptomatic. MRI scans show an adjacent segment stenosis at level L3/4. Furthermore, long standing x-ray imaging and CT scans of the patient are provided.

Median age of the patients was 73 (62–78) years. A total of 86.5% of patients suffered from a proximal adjacent segment stenosis and most common level was L3/4 (51.4%). A total of 62.2% of included patients developed adjacent segment stenosis after fusion of one single lumbar segment. Eighteen patients were included in MDG and 19 patients in FG. All patients of the MDG were treated via laminectomy and undercutting. Sixteen patients of the FG were treated via laminectomy and undercutting, 2 patients with hemilaminectomy, and 1 patient with laminectomy. A total of 56.8% of the patients had one prior lumbar operation and 62.2% one fusioned segment. Data show a severe disability in activities of daily life of the included patients with a median of ODI of 56 (36–61). Overall median PSQI of sleep quality was 10 (6–15), indicating an overall preoperative severe poor sleeping behavior. Physical Scale of Quality-of-life assessment via SF-36 showed a Physical Component Summary Score (PCS) of 28.3 (27.0–30.7) and a Mentally Health Component Summary Score (MSC) of 30.9 (18.4–44.9) of the study population. Mood state analysis showed a median of ADS-K depression score of 16 (8–20). Concerning radiographical determined parameters, the two groups showed no significant differences. All patients with overt instability in long standing and flexion/extensions studies were excluded from the study. Detailed baseline characteristic of the total study population stratified into MDG and FG are displayed in Table 1.

**Table 1:** Baseline characteristics and stratification regarding decompression with or without fusion

	Total study population (*n* = 37)	MDG (*n* = 18)	FG (*n* = 19)		*p*-value
Age, yr, median (IQR)	73 (62–78)	75 (59–79)	69 (63–77)	n.s. (0.425)	
Female sex, *n* (%)	23 (62.2)	12 (66.7)	11 (57.9)	n.s. (0.582)	
BMI, kg/m^2^, median (IQR)	27.7 (24.3–33.5)	28.8 (24.5–34.7)	27.6 (22.5–32.8)	n.s. (0.324)	
Cranial adjacent level stenosis, yes, *n* (%)	32 (86.5)	17 (94.4)	15 (78.9)	n.s. (0.168)	
Level of adjacent stenosis				sig. (0.039)	
LWK1/2	2 (5.6)	1 (5.6)	1 (5.3)		
LWK2/3	9 (25.0)	6 (33.3)	3 (15.8)		
LWK3/4	19 (51.4)	9 (50.0)	10 (52.6)		
LWK4/5	4 (11.1)	2 (11.1)	2 (10.5)		
LWK5/SWK1	3 (8.3)	0 (0.0)	3 (15.8)		
Amount of lumbar preoperations				n.s. (0.068)	
1	21 (56.8)	7 (38.9)	14 (73.7)		
2	10 (27.0)	8 (44.4)	2 (10.5)		
3	5 (13.5)	3 (16.7)	2 (10.5)		
4	1 (2.7)	0 (0.0)	1 (5.3)		
Number of preoperative fusioned segments				n.s. (0.138)	
1	23 (62.2)	14 (77.8)	9 (47.4)		
2	12 (33.3)	4 (22.2)	8 (42.1)		
3	2 (5.6)	0 (0.0)	2 (10.5)		
Time from initial fusion to adjacent stenosis, months, median (IQR)	52 (15–107)	52 (15–129)	53 (14–104)	n.s. (0.616)	
Preoperative radiographic measurements					
Lumbar Lordosis, °, median (IQR)	36 (27–50)	35 (26–50)	36 (28–51)	n.s. (0.817)	
Pelvic incidence, °, median (IQR)	58 (50–65)	58 (47–65)	58 (53–66)	n.s. (0.683)	
Segment lordosis at fusioned level, °, median (IQR)	19 (14–28)	21 (15–28)	18 (12–28)	n.s. (0.444)	
HU of adjacent vertebral body, °, median (IQR)	135 (95–186)	150 (88–209)	135 (96–182)	n.s. (0.788)	
Disc height, mm, median (IQR)	8 (6–9)	8 (6–8)	8 (6–9)	n.s. (0.474)	
Minimal diameter of spinal canal, median (IQR)	4.7 (2.7–5.6)	4.4 (3.6–4.4)	4.7 (2.2–7.3)	n.s. (0.191)	
Preopoperative functional scores					
Pain at rest, NRS score, median (IQR)	5 (4–7)	5 (4–7)	6 (4–8)	n.s. (0.692)	
Pain in motion, NRS score, median (IQR)	8 (8–9)	8 (8–9)	8 (8–9)	n.s. (0.274)	
ODI score (%), median (IQR)	56 (36–61)	55 (26–59)	58 (37–62)	n.s. (0.297)	
PSQI score, median (IQR)	10 (6–14)	10 (4–13)	12 (8–14)	n.s. (0.599)	
ADS-K depression score, median (IQR)	16 (8–20)	14 (8–25)	17 (12–19)	n.s. (0.945)	
SF-36 PCS, median (IQR)	28.4 (27.0–30.7)	28.3 (23.0–30.1)	28.3 (27.8–30.8)	n.s. (0.450)	
SF-36 MCS, median (IQR)	30.9 (18.6–44.9)	32.0 (27.5–51.2)	18.7 (15.6–36.7)	n.s. (0.223)	

Abbreviations: *MDG* microsurgical decompression group, *FG* gusion group, *ADS* General Depression Scale, *HU* Hounsfield unit, *IQR* interquartile range, *NRS* numerical rating scale, *n* number, *ODI* Oswestry disability Index, *PSQI* Pittsburgh Sleep Quality Index, *yr* years, *n.s*. not significant, *sig*. significant

### Outcome

Ten patients, 5 (2 surgical complications and 3 spine revision surgery) in MDG vs. 5 (1 surgical complication and 4 spinal revision surgery) in FG (*p* = 0.920), had a need for a further lumbar surgery within the first year after the intervention. Therefore, the primary outcome of the study shows no difference between the two groups. Detailed reasons for the interventions are provided in Fig. 2. Moreover, further follow studies with a median time from surgery of 30 months (19–45) showed no change between the need for further lumbar surgery (6 in MDG vs. 7 in FG, *p* = 0.823). However, follow-up time in the FG is longer than in MDG.

**Fig. 4:**
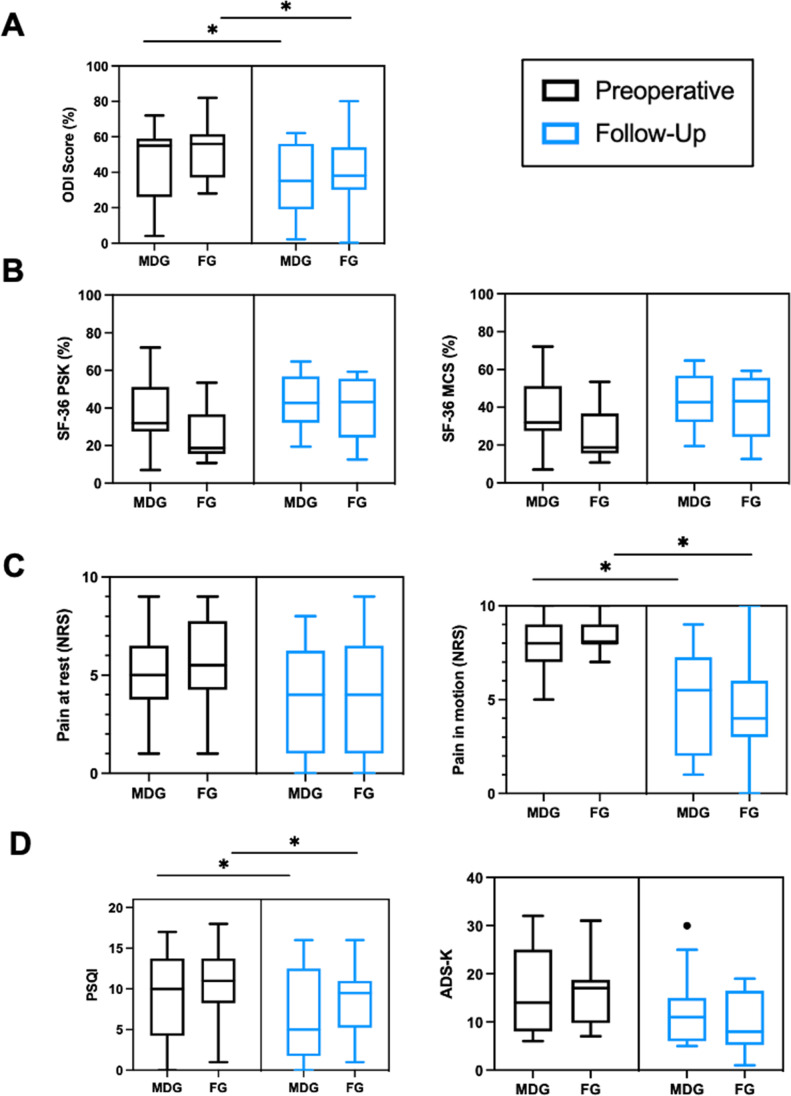
Functional outcome of patients after surgery. ODI: 0, minimal disability — 100, maximum disability; SF-36 (0, maximal limitations — 100, minimal limitations). PSQI: 0, no difficulty in sleeping behavior — 21, severe difficulties. ADS-K: ADS-K ≥ 17, depressive disorder, *p < 0.05

Both, MDG and FG benefited from surgical decompression and showed similar results concerning the outcome scores after 1 year. In detail, median of pain associated disability index decreased significantly in both groups from severe disability to moderate disability (*MDG*: *p = 0.048, *FG*: *p = 0.01). Pain on motion significantly improved in both groups (*MDG*: *p = 0.02, *FG*: *p = 0.01). Mentally Health and Physical Scale of Quality of life and General Depression Scale showed positive trends in both intervention groups. Median sleeping quality index PSQI improved significantly from chronic sleeping disorder to bad sleeping (*MDG*: *p = 0.014, *FG*: *p = 0.026). An overview about functional outcome parameters prior surgery and 1 year after surgery is provided in Fig. 4.

Duration of surgery (*p < 0.001) and hospital stay (*p = 0.04) were significantly shorter in the MDG compared to the FG. Detailed operative and functional outcomes of total study population and MDG and FG are presented in Table 2.

**Table 2:** Operative and functional outcome of patients and stratification regarding decompression with or without fusion

	Total study population (*n* = 37)	MDG (*n* = 18)	FG (*n* = 19)		*p*-value
Number of targeted levels				sig. (0.039)	
1	33 (89.2)	18 (100.0)	15 (78.9)		
2	4 (10.8)	0 (0.0)	4 (21.1)		
Dura leakage, yes, *n* (%)	2 (5.4)	2 (11.1)	0 (0.0)	n.s. (0.135)	
Duration of surgery, min, median (IQR)	139 (82–12)	82 (60–98)	211 (158–252)	sig. (< 0.001)	
Duration of hospital stay, d, median (IQR)	5 (4–10)	4 (3–6)	6 (4–14)	sig. (0.006)	
Clinical follow-up after 1 year					
Pain at rest, NRS score, median (IQR)	4 (1–6)	4 (2–6)	4 (1–7)	n.s. (0.723)	
Pain in motion, NRS score, median (IQR)	4 (3–7)	6 (2–7)	3 (4–6)	n.s. (0.580)	
ODI score (%), median (IQR)	39 (26–55)	35 (19–56)	40 (31–53)	n.s. (0.551)	
PSQI score, median (IQR)	6 (4–11)	5 (2–13)	10 (5–11)	n.s. (0.302)	
ADS-K depression score, median (IQR)	10 (6–17)	12 (7–17)	8 (5–17)	n.s. (0.280)	
SF-36 PCS, median (IQR)	32.7 (28.0–40.9)	30.0 (25.9–40.0)	34.0 (29.8–41.4)	n.s. (0.205)	
SF-36 MCS, median (IQR)	42.9 (31.6–55.6)	41.2 (31.6–56.4)	43.2 (24.3–55.6)	n.s. (0.909)	
Need for further lumbar surgery, yes, *n* (%)	10 (27.0)	5 (27.8)	5 (26.3)	n.s. (0.920)	
Second follow-up					
Follow-up time, months, median (IQR)	30 (19–45)	19 (16–35)	38 (23–51)	sig. (0.002)	
Need for further lumbar surgery, yes, *n* (%)	13 (35.1)	6 (33.3)	7 (36.8)	n.s. (0.823)	

Abbreviations: *MDG* microsurgical decompression group, *FG* fusion group, *ADS* General Depression Scale, *HU* Hounsfield unit, *IQR* interquartile range, *NRS* numerical rating scale, *n* number, *ODI* Oswestry disability Index, *PSQI* Pittsburgh Sleep Quality Index, *yr* years, *n.s*. not significant, *sig*. significant

## Discussion

The principal novel finding of the study is that decompression may not be inferior to decompression and fusion in patients suffering from degenerative adjacent segment stenosis without obvious signs of instability, deformation, and neuroforaminal stenosis after lumbar fusion in short-term follow-up. Due to significant shorter time of the surgery, a pure microsurgical decompression may be a sufficient alternative to a decompression and fusion, particular regarding old age of the patients collective. Therefore, it is tempting to treat this patient collective as patients with primary spinal stenosis. However, long-term results are pending and will clarify the need for further fusion after decompression of adjacent spinal stenosis.

Spinal fusions are standard methods for surgical treatment of deformity, trauma, and degenerative disorders [15]. Thereby, degenerative adjacent segment stenosis is a common long-term complication with an incidence of symptomatic patients ranging from 5 to 19% [5]. Exact mechanisms of origin are still uncertain. Biomechanical studies showed a change of mechanical stress and loading on the adjacent segments. In contrast, other studies suggest that adjacent stenosis might be the result of a natural degenerative process [5, 15–17]. Among others, age, BMI, history of smoking, preoperative adjacent disc degeneration, and long-segment fusion are considered risk factors for the development of adjacent stenosis after spinal fusions [3]. Adjacent segment stenosis to previous lumbar fusion can lead to significant discomfort and pain [8, 18]. If further therapy is required, current literature is providing only limited data concerning surgical treatment of this entity and resulting clinical outcomes. Potential strategies can be decompression, decompression with fusion, or even corrective surgery. It is still uncertain whether patients with adjacent spinal stenosis without obvious instability, neuroforaminal stenosis, or deformity should be treated via decompressive surgery alone or decompression and fusion. Therefore, the choice of the operative technique remains still an individual decision. In primary symptomatic lumbar spinal stenosis decompression alone, surgery is leading to improvement in pain and function [19]. Whitecloud et al. [20] reported about 12 patients with a median age of 53 years suffering from stenotic adjacent stenosis after previous lumbar fusion treated with decompression and fusion. Detailed follow-up time was not provided. None of the patients had an excellent result, indicating no symptoms except for occasional back pain, no medications required, and return to work. Six of the 12 patients reported some improvement, further need of pain medication, and functional restriction. Three patients reported no change or worsening of the symptoms after fusion. Schlegel et al. [21] show the 2 years follow-up data of 37 patients who were surgically treated (23 with decompression, 14 patients with decompression and fusion) after previously thoracolumbar or lumbar fusions. Patients with adjacent segment diseases were included (including spondylolisthesis, stenosis, herniated nucleus pulposus, kyphoscoliosis, spinal fractures) and mean age was 43 years. A total of 70.3% of these patients reported a good or excellent outcome. Seven patients (18.9%) required an additional surgical procedure during the follow-up period. Philipps et al. [8] provide follow-up data (mean of follow-up 5 years after decompression) of 26 patients with a median age of 54 years suffering from spinal stenosis at the lumbar segments adjacent to a previous lumbar fusion. All patients were treated with decompression only. Fifteen patients rated their outcome as completely satisfactory, 5 considered their surgery as failure. Six patients (23%) required a further lumbar surgery during the follow-up period. This is in range with the data of the current study (MDG after 1.5 years: 29.4%).

The present data show that patients with adjacent spinal stenosis due to prior lumbar fusion are not only suffering from the physical disability and pain but also from psychiatric burden and sleeping disorders. Mean of Physical SF-36 Component Summary Score in German population with an age from 70 to 79 years is 42.5 for women and 42.4 for men. SF-36 Mentally Health Component Summary Score in this cohort is 50.1 for women and 51.0 for men [22]. Preoperative median PCS score of 28.4 (27.0–30.7) and MCS score of 30.9 (18.6–44.9) are indicating considerable levels of suffering of the study population. This is underling the clinical need for sufficient therapies for this patient’s cohort.

At short-term follow-up, this study shows no difference concerning clinical outcome between both surgical groups. Both groups improved significantly in pain associated disability index, pain in motion, and sleeping quality and show positive trends in all other clinical outcome parameters. These clinical improvements are in line with short-term outcomes after decompression of primary symptomatic spinal stenosis as investigated in the SPORT-trial [19]. This empowers the basic strategy to treat symptomatic adjacent stenosis with no neuroforamen stenosis, deformity, and/or instability, like primary symptomatic spinal stenosis. With a median age of 72 years of the study population, the results underline that especially elderly patient suffer from adjacent segment stenosis. Considering possible age-related comorbidities and higher preoperative risks, the significant shorter time of surgery and hospital stay are advantages of the smaller “decompression only” surgery that should not be underestimated.

This study is inherently limited due to its retrospective study design and the small sample size. It is underpowered to show a potential non-inferiority of the pure decompression vs. decompression with fusion. Larger, prospective studies are needed to verify our results. Furthermore, this study provides only data about short-term follow-up, and long-term results are pending. To create further evidence, it is planned to prospectively monitor the study population and to prepare a prospective controlled trial. Some patients reported mentally discomforted due to the current corona pandemic situation. This is a potential bias in the 1-year follow-up regarding to depression and mentally health scores.

## Data Availability

Not applicable.
